# An economic evaluation for prevention of diabetes mellitus in a developing country: a modelling study

**DOI:** 10.1186/1471-2458-13-729

**Published:** 2013-08-07

**Authors:** Xiaoqian Liu, Changping Li, Hui Gong, Zhuang Cui, Linlin Fan, Wenhua Yu, Cui Zhang, Jun Ma

**Affiliations:** 1 Department of Health Statistics, College of Public Health, Tianjin Medical University, No. 22 Qixiangtai Road, Heping District, Tianjin 300070, P.R. China; 2 Institute of New Catalytic Materials Science, College of Chemistry, Nankai University, Tianjin 300071, P.R. China

## Abstract

**Background:**

The serious consequences of diabetes mellitus, and the subsequent economic burden, call for urgent preventative action in developing countries. This study explores the clinical and economic outcomes of strategies that could potentially prevent diabetes based on Chinese circumstances. It aims to provide indicators for the long-term allocation of healthcare resources for authorities in developing countries.

**Methods:**

A representative sample of Chinese adults was used to create a simulated population of 20,000 people aged 25 years and above. The hybrid decision tree Markov model was developed to compare the long-term clinical and economic outcomes of four simulated diabetes prevention strategies with a control group, where no prevention applied. These preventive strategies were the following: (i) one-off screening for undiagnosed diabetes and impaired glucose tolerance (IGT), with lifestyle interventions on diet, (ii) on exercise, (iii) on diet combined exercise (duo-intervention) respectively in those with IGT, and (iv) one-off screening alone. Independent age-specific models were simulated based on diverse incidences of diabetes, mortalities and health utilities. The reported outcomes were the following: the remaining survival years, the quality-adjusted life years (QALYs) per diabetes or IGT subjects, societal costs per simulated subject and the comparisons between preventions and control over 40 years. Sensitivity analyses were performed based on variations of all assumptions, in addition to the performance and the compliance of screening.

**Results:**

Compared with the control group, all simulated screening programmes prolonged life expectancy at the initiation ages of 25 and 40 years, postponed the onset of diabetes and increased QALYs at every initiation age. Along with an assumption of six years intervention, prevention programmes were associated with cost-saving compared with the control group, especially in the population aged 25 years. The savings were at least US$2017 per subject, but no statistically significant difference was observed among the intervention strategies within each age groups. The cost savings were reduced when screening was affected by poor performance and noncompliance.

**Conclusions:**

Developing countries have few effective strategies to manage the prevention of diabetes. One-off screening for undiagnosed diabetes and IGT, with appropriate lifestyle interventions for those with IGT are cost saving in China, especially in young adults.

## Background

Diabetes mellitus is expected to become one of the most serious health problems in the world within the next 25 years [1]. The number of patients with diabetes has increased dramatically in developing countries [2], especially in China. Currently, China has the largest diabetes population among developing nations with approximately 92.4 million adults aged 20 years or older, of which 60.7% are undiagnosed [3]. In China, the direct medical costs of diabetes and related complications is estimated to have been US$26.0 billion in 2007, which represented 81% of total medical care costs [4]. These costs are expected to increase to $47.2 billion by 2030. In addition, direct health expenditure on diabetes accounted for 18.2% of total government annual health expenditure in 2007, which was much higher than that of developed countries [5], such as Spain with 7.4% [6], USA with 11.9% [7] and Germany with 14.2% [8]. The medical and economic burden of diabetes is an important public health challenge for China. However, at present, there is no systematic prevention strategy for diabetes, despite the fact that it is considered a fundamental component of the nation’s health policies [9].

Screening for undiagnosed diabetes and pre-diabetes could provide early diagnosis and could allow for medical treatment to start, thus slowing down the progress of the disease. Another effective approach is lifestyle intervention, which, over the last century, has been shown to result in a dramatic decrease in the incidence of diabetes in both developed and developing countries [10-17]. However, it is not certain whether such strategies should be brought together and implemented in developing countries with their limited health resources, since no reported evaluations exist in countries like China to show whether these prevention strategies are economically viable.

At present, there have been no clinical trials evaluating the economic effects of screening for pre-diabetes at various ages, with or without interventions. Most recommendations for non-pharmacological preventions of diabetes have been based on mathematical models [13,18-29]. In most of these models, favourable results have been generated by simulating screening or intervention strategies alone, and these strategies have been carried out in high-income areas, rather than in developing countries. There has been one study that compared screening followed by interventions with no screening [26]. Unfortunately, this study did not consider an appropriate initiation age for the prevention strategies.

To address the above issues, we collected data from high-quality studies that involved the detection and prevention of diabetes in China. Based on these studies, a hybrid decision tree Markov model was performed to estimate the clinical and economic outcomes of screening for undiagnosed diabetes and impaired glucose tolerance (IGT), followed by the implementation of lifestyle intervention in those with IGT. By comparing these results with no screening, the dominant strategies and targeted populations were selected. It is hoped that these findings will provide a model for authorities of developing countries to optimise the allocation of health resources.

## Methods

Selected model inputs and assumptions are shown in Tables 1 and 2. Estimated parameters, prevention and treatment costs, and health-related quality of life weight (also called utility score or utility) were obtained from published data. The screening unit cost was derived from Publicity Medicine Prices of Hunan, China.

**Table 1:** Baseline values of input parameters used in models (Epidemiology parameters and costs (US$))

**Parameters**	**Screening with diet intervention**	**Screening with exercise intervention**	**Screening with duo-intervention**	**Screening alone**	**Control**	**References**
**Epidemiology parameters**						
Negative rate of 2-h PG^¢^	0.96	0.96	0.96	0.96	−	[30]
Positive rate of OGTT^£^	0.305	0.305	0.305	0.305	−	[30]
Proportion of Diagnosed IGT^¤^	0.478	0.478	0.478	0.478	−	[30]
Normal PG to IGT^§^	0.0128	0.0128	0.0128	0.0128	0.0128	[30,31]
IGT to onset of diabetes^¶^						
Initiation age of 25	0.0290	0.0273	0.0275	0.0400	0.0644	[12,32,33]
Initiation age of 40	0.0754	0.0710	0.0716	0.104	0.1670	[12,32]
Initiation age of 60	0.2320	0.2184	0.2200	0.3600	0.5778	[12,32,33]
IGT to normal PG^ß^	0.116	0.116	0.116	0.116	0.116	[34]
Onset of diabetes to CVD^ð1^	0.062	0.062	0.062	0.0675	0.0675	[35,36]
Onset of diabetes to Nephropathy^ð2^	0.001	0.001	0.001	0.001	0.001	[35,36]
Onset of diabetes to Neuropathy^ð3^	0.0043	0.0043	0.0043	0.005	0.005	[35,36]
Onset of diabetes to Retinopathy^ð4^	0.0046	0.0046	0.0046	0.0081	0.0081	[35,36]
CVD to death^&1^	0.0058	0.0058	0.0058	0.0087	0.0087	[35,36]
Nephropathy to death^&2^	0.0008	0.0008	0.0008	0.0003	0.0003	[35,36]
**Costs (US$)**^**a**^						
Cost for screening	3	3	3	3	−	[37]
Diet or exercise intervention	362	362	−	−	−	[38]
Duo-intervention	−	−	371	−	−	[38]
Onset of diabetes	897	897	897	897	897	[5]
CVD treatment	2078	2078	2078	2078	2078	[5]
Nephropathy treatment	1089	1089	1089	1089	1089	[5]
Neuropathy treatment	1324	1324	1324	1324	1324	[5]
Retinopathy treatment	888	888	888	888	888	[5]

**Figure 1:**
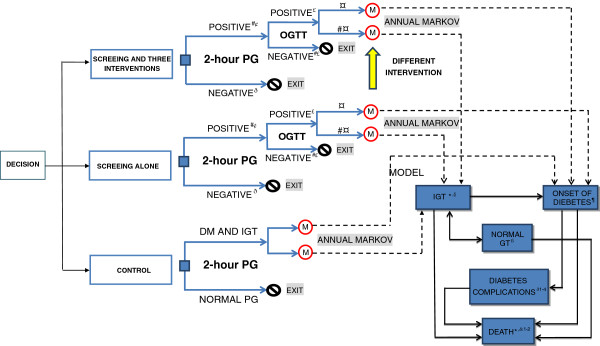
**Progression of individuals screened and intervened for diabetes.** The hybrid tree combined a decision tree and Markov models. The decision tree (the left side) consisted of five main arms representing five scenarios. The first three scenarios involved screening for undiagnosed diabetes and IGT followed by any of the three active lifestyle interventions (diet, exercise, and duo-intervention), which were applied to the IGT subjects. The fourth scenario involved screening for undiagnosed diabetes and IGT, but without formal interventions, and the fifth scenario involved control group. Nine Markov models represented the nature history of diabetes (the lower right side). Each of them consisted of eight states: IGT, normal glucose tolerance, onset of diabetes, four diabetes complication states and death. The IGT states were tunnel states that included six temporary ones representing 6 years lifestyle interventions. Transition probability, costs, benefits were required for each state. Three separate models were performed for strategies starting at age of 25, 40 and 60 respectively. “2-hour PG” means 2-hour plasma glucose after breakfast. “DM” means diabetes mellitus. “OGTT” means oral glucose tolerance test. “IGT” means impaired glucose tolerance. “NORMAL GT” represented normal glucose tolerance state. “DIABETES COMPLICATIONS” included four different diabetes complications states: cardiovascular disease, retinopathy, nephropathy, and overt neuropathy disease. We numbered the transition paths corresponding to the main transition parameters in Table 1. (^#^The complement probabilities of one branch. *The life-table information used to model competing causes of death. ^@^The proportion of individuals with normal PG. ^¢, £, ¤^ Transition parameters which determined whether a subject would receive interventions. ^§, ¶, ß, ð,^^&:^ Transition parameters applied to the Markov models: ^ð1^ to ^ð4^ determined transitions from onset of diabetes state to complications states respectively; ^&1^ and ^&2^ determined transitions from CVD or nephropathy to death state. We did not include the neuropathy-specific and retinopathy-specific mortalities, since these complications are not fatal).

The hybrid tree combined a decision tree and Markov models. The decision tree consisted of five main arms representing five scenarios. The first three scenarios involved screening for undiagnosed diabetes and IGT followed by any of the three active lifestyle interventions (diet, exercise, and duo-intervention), which were applied to the IGT subjects. The fourth scenario involved screening for undiagnosed diabetes and IGT, but without formal interventions (physicians dispensed information brochures only), and the fifth scenario involved control group with no screening or intervention. ‘**OGTT**’ means oral glucose tolerance test. ‘**IGT**’ means impaired glucose tolerance. ‘**PG**’ means plasma glucose. ‘**CVD**’ means cardiovascular disease. Costs(US$)^a^ involved following costs: unit costs of one-off screening (test of 2-hour PG after breakfast, a confirmatory OGTT), and were derived from the national standard prices of medicines in China; costs of lifestyle interventions (diet or exercise intervention, and duo-intervention) were the average annual costs and obtained from a domestic community-based trial in China[38]; treatment costs of diabetes-related disorders were derived from a study of treatment costs for diabetes in China [5]. The transition parameters were numbered corresponding to the transition paths in Figure 1. (^#^The complement probabilities of one branch. *The life-table information which was used to model competing causes of death. ^@^The proportion of individuals with normal PG. ^¢, £, ¤^ Transition parameters which determined whether a subject would receive interventions. ^§, ¶, ð,^^&:^ Transition parameters used in the Markov models, among these, ^ð1^ to ^ð4^ determined four transitions from onset of diabetes state to different complications states (cardiovascular disease, retinopathy, nephropathy and overt neuropathy) respectively; ^&1^ and ^&2^ determined two transitions from CVD or nephropathy state to death state. We did not consider the neuropathy-specific and retinopathy-specific mortalities, since these complications are not fatal).

**Table 2:** Utilities assigned to various health states of Markov models at different initiated ages of prevention

**Health states**	**Intiation ageof 25**	**Intiation ageof 40**	**Intiation ageof 60**	**References**
Normal PG	1	1	1	−
IGT	0.95	0.95	0.95	[19]
Onset of diabetes	0.805	0.800	0.794	[39,40]
CVD	0.679	0.674	0.584	[39,40]
Retinopathy	0.705	0.700	0.610	[39,40]
Nephropathy	0.646	0.641	0.551	[39,40]
Neuropthy	0.667	0.662	0.572	[39,40]
Death	0	0	0	−

The unadjusted median utilities for diabetes-related disorders represented the median age and median social demography of individuals having diabetes, thus, these values were assigned to individuals aged 40 years [39,40]. By using the coefficient of age and three disorder conditions to adjust the baseline estimate of initiation age at 40, the utilities of 60-year-old subjects with a complication were determined. For example, the utility of subjects with diabetes having CVD at initiation age of 60 was 0.584. It was derived from the median utility for diabetes with CVD (0.674) subtracting 0.006 ((60–40)*(−0.0003)) and 0.084 (coefficient of three conditions included diabetes with CVD). For subjects aged 25 years, the calculation of utilities only considered the impact of age (coefficient of age was used to adjust the baseline estimates of aged 40 years) [40].

### Study design

Because there are no primary prevention policies being generally implemented in developing countries, we built a hybrid model, which was constructed by a decision tree and Markov models, to simulate potential screening and lifestyle intervention strategies for the prevention of diabetes (Figure 1). The decision tree included five main arms representing five scenarios. The first three scenarios involved screening for undiagnosed diabetes and IGT followed by one of the three active lifestyle interventions (diet, exercise or duo-intervention), which were applied to the IGT subjects. The fourth scenario involved screening for undiagnosed diabetes and IGT, but without the formal lifestyle interventions. The fifth scenario involved the control group with no screening or intervention. The simulated individuals were subjected to one of the five different strategies in sequence.

The decision tree used positive screening rates and the prevalence of diabetes and IGT in the reference population to determine how many individuals started in each state of the Markov models. Each Markov model consisted of eight main health states: IGT, normal glucose tolerance, onset of diabetes, four diabetes complication states and death. Among these, the IGT states contained subjects who had a 2-h plasma glucose (PG) concentration between 6.7 and 11.1 mmol/L. These states were tunnel states that included six temporary ones representing 6 years of lifestyle intervention. The simulated IGT subjects underwent the tunnel states until they reached either normal glucose tolerance, onset of diabetes or death. The normal glucose tolerance state applied to subjects who had a 2-h PG <6.7 mmol/L. The onset of diabetes state applied to subjects who had a 2-h PG >11.1 mmol/L [30]. There were four diabetes complication states: cardiovascular disease (CVD), including myocardial infarction or stroke [35], retinopathy, including proliferative retinopathy, photocoagulation or blindness, nephropathy, including end-stage renal disease or renal replacement, and overt neuropathy, including abnormal touch sensation, ulceration or extremity amputation [36]. The last state, death, contained subjects who died from natural causes or from diabetes.

The Markov models ran for a time horizon of 40 years, and each of the model cycles represented 1 year. Separate simulations with different incidence rates of diabetes, mortality rates and health utilities were performed for the diabetes prevention programmes or for the control starting at 25, 40 and 60 years, respectively.

The hybrid model parameters were estimated by using Markov chain Monte Carlo simulation methods [28], and results were derived from a sample of 20,000 simulations. The main outcomes analysed in the model were the remaining survival years and the health effectiveness QALYs per subject with diabetes or IGT, the life-years gained before the onset of diabetes or before the onset of any kind of complication per subject with IGT and the cost per subject for prevention strategies or control at the different initiation ages.

### Screening and interventions

The subjects with 2-h PG ≥6.67 mmol/L after breakfast were given an oral glucose tolerance test (OGTT) [30]. The IGT subjects diagnosed from screening received different types of lifestyle interventions [12]. The diet group were encouraged to consume more vegetables and to control their intake of alcohol and simple sugars. The exercise group were taught and encouraged to increase their leisure physical activity, and the duo-intervention groups were given information regarding both diet and exercise. Participants in these three groups received individual counselling from physicians or the counselling sessions. These interventions continued for six years with an average of nine sessions a year. The group that had screening alone only received information brochures with general instructions on diet or exercise from clinic physicians [12].

### Epidemiological variables

Data from a population-based cross-sectional study of 110,660 residents aged 25–74 years in China were used to provide representative population characteristics, screening methods and screening positive rates [30]. The annual incidence of IGT was deduced from the prevalence rate of IGT [30] and the average time before diagnosis of diabetes [31]. The annual transition rate from the IGT state to normal glucose tolerance was calculated using a 3-year cumulative incidence of a spontaneous return to normal glucose tolerance [34]. Based on data from prospective diabetes intervention studies in China, estimates of the incidences and mortalities of the four diabetes complications (cardiovascular disease, retinopathy, nephropathy and overt neuropathy) varied with the different strategies used [35,36].

The different transition rates from the state of IGT to onset of diabetes were used to reflect the natural history of diabetes at different ages. For the study initiation age of 40 years, the baseline transition rates for the prevention groups were obtained from the cumulative incidence of diabetes at the sixth year [12], and a 3-year cumulative incidence of diabetes was used as the control [32]. By using the ratio of diabetes incidence at ages 25, 40 and 60 years to adjust the baseline estimates at age 40 years, the transition rates from IGT to onset of diabetes state for the initiation ages of 25 and 60 years were calculated. The ratio was approximately 1:2.6:8 according to Dunstan et al. [33].

The life-table information was used to evaluate the competing causes of death at the different initiation ages [41].

### Economic variables

The costs of all expenditure relating to diabetes prevention and treatment were collected from a societal perspective, including direct medical, direct nonmedical and indirect costs [42]. Generally, the direct medical costs were co-payment fees for treatment, diagnostic testing, prescription drugs and medical supplies. Direct nonmedical costs related to expenses for services like transportation for the patient and family members to clinics. The lost income of the patients and their families as well as the costs for hiring nurses or care providers were regarded as indirect costs [5].

Screening costs were the laboratory expenses that included the cost of the initial 2-h PG screening test after breakfast, and the confirmatory diagnostic OGTT in subjects who had a positive PG test [37]. Costs assigned to subjects in the IGT states included those from the lifestyle interventions, such as the costs of increased visits to the general practitioners and the counselling sessions [38]. Costs for onset of diabetes state were submitted by the patients without complications, while average costs, which were applied to the diabetes complication states, were calculated by dividing the total costs by the number of patients with corresponding complications [5].

The costs in Chinese yuan were converted to US dollars using the exchange rate as of June 15, 2007 (US$1 = CHY￥7.6948).

### Quality-of-life variables

QALYs of subjects were calculated according to the time spent with the health states and the utilities assigned to these states [43]. For individuals with normal glucose tolerance and IGT, the utilities were determined as 1 and 0.95, respectively [19]. The unadjusted median utilities for diabetes-related disorders were assigned to the subjects at age 40 years [39,40], because these values represented the median age and median social demography of individuals having diabetes. The utilities of subjects aged 25 and 60 years were calculated based on the age-related characteristics of diabetes. In contrast to the younger subjects with diabetes, the older subjects had one more universal coexisting condition [44]. Thus, using the coefficient of age and three coexisting conditions (including diabetes and a given complication), the utilities of the 60-year-old subjects with a complication were determined by adjusting the baseline estimates of subjects aged 40 years. For example, the utility of the subjects with diabetes having CVD at the initiation age of 60 years was 0.584, which was derived from the median utility of diabetes with CVD (0.674) and subtracting 0.006 [(60–40) × (−0.0003)] and 0.084 (the coefficient of three conditions). For the individuals aged 25 years, the calculation of utilities only considered the impact of age [40], because no differences in severity of disease have been found between young and the middle-aged subjects with diabetes [45].

Costs and QALYs were discounted at the rate of 3% [18]. Half-cycle corrections for both costs and health effects were applied to the model [28].

### Sensitivity and statistical analysis

Sensitivity analyses were based on a change of one parameter at a time. All parameters related to the performance of screening, prevalence of disease, costs, utilities and discount rates were studied using sensitivity. Some of the main assumptions were increased or decreased by 20%. These included positive rates of screening, incidence of diabetes, incidence or mortality of diabetes-related disorders, costs of screening, interventions and treatment of diabetes-related disorders, and utilities associated with a range of health states. In particular, the assumptions concerning the incidence of IGT were increased by 200% and decreased by 50%, since the difference in prevalence of IGT between young and old individuals is large in China, almost 200% [3]. The sensitivity analyses of the detection level of IGT from screening at 80% and 60% were performed. For the compliance level of screening, 80% and 60% were also performed. Changing the model inputs allowed us to evaluate the robustness of the model.

The statistical analyses were performed with SAS 9.0 (SAS Institute Inc., Cary, NC, USA). The model simulations were done with the software TreeAge 2011 Software (Williamstown, MA, USA). Continuous outcomes were compared by use of the Wilcoxon rank-sum test. Results with a p-value of <0.05 were considered significant.

## Results

Based on the hybrid decision tree Markov model, the remaining survival years was 30.7 (19.8–41) for screened subjects with diabetes and subjects with IGT who received any of the three active lifestyle interventions (diet, exercise or duo-intervention) using a start age of 25 years. For screening alone, it was 30.2 (18.7–40.5) and 29.0 (18.5–40.3) years for the control. The remaining life-years gained that were induced by the prevention strategies ranged from 1.2 to 1.7 years compared with the control. With respect to the subjects with diabetes or IGT at the initiation age of 40 years, the remaining survival years for screening combined with the three lifestyle intervention strategies was 20.1 (10.8–29.4) years, for screening alone it was 19.7 (10.4–29) years and for the control it was 19.6 (10.5–28.4) years. The remaining survival life-years gained from interventions and from screening alone were approximately 0.5 and 0.1 years, respectively. When the initiation age went up to 60 years, the remaining survival years went down to 7.5 (2.6–12.4) for the intervention strategy groups and 7.4 (2.5–12.4) for screening alone and for the control. In this case, intervention strategies produced approximately a 0.1 year longer lifetime than with screening alone and with the control. A significant difference of survival years was found between the three lifestyle intervention strategies and the other two groups at the initiation ages of 25 and 40 years, but not at 60 years.

Compared with the control, the IGT subjects who received prevention strategies gained more years before having diabetes or of any kind of diabetes-related complication. We used the term extra time for these years. Based on Table 3, all prevention strategies, except screening alone at the initiation age of 60 years, prolonged the time before onset of diabetes, and also deferred the time of onset of diabetes-related complications, i.e. extra time was offered by preventions (4.48–5.20 years at age 25 years, 2.68–3.06 years at age 40 years, and less than 1 year at age 60 years). Additionally, the initiation ages were found to show a proportional effect on extra time.

**Table 3:** The extra time gained for individuals with IGT before developing diabetes related disorders by preventions at different initiation ages

**Different strategies**	**Years gained before development of diabetes onset**			**Years gained before development of complications**		
	**Age 25**	**Age 40**	**Age 60**	**Age 25**	**Age 40**	**Age 60**
Screening with diet intervention	2.51	1.59	0.49	4.48	2.68	0.68
Screening with exercise intervention	2.94	1.85	0.57	5.20	3.06	0.78
Screening with duo-intervention	2.88	1.81	0.55	5.11	3.00	0.75
Screening alone	0.04	0.01	0	0.08	0.02	0

Subjects with IGT who received prevention strategies gained more years before having diabetes and of any kind of complications than the control, the term extra time was used for these years. All prevention strategies, except screening alone at initiation age of 60 years, offered some extra time. Moreover, the initiation ages were found to show proportional effect on extra time.

The results showed that both the screening with interventions and screening alone groups increased QALYs of subjects either with diabetes or IGT compared with the control (p <0.0001 within each age group) (Table 4). Also, the increase in QALYs was considerably lower for all prevention strategies at 60 years of age. Therefore, the younger the age at screening, the more benefits were achieved.

**Table 4:** The clinical and economic outcomes of prevention strategies and control (or compared with control) for subjects at different initiation ages

	**Screening with diet intervention**	**Screening with exercise intervention**	**Screening with duo-intervention**	**Screening alone**	**Control**
**Initiation age of 25**					
Remaining life years^I^	30.7	30.7	30.7	30.2	29.0
Costs (US$)^II^	13294.77	13234.20	13241.38	18973.08	20102.87
	(2750.37 - 27317.10)	(2744.20 - 27306.59)	(2745.42 - 27311.41)	(3755.50 - 33613.01)	(5334.50 - 37291.10)
Saving costs(US$)^III^	6808.10	6868.67	6861.49	1129.79	−
QALYs^IV^	17.98	17.98	17.98	17.05	14.65
	(13.00 - 21.40)	(13.10 - 23.40)	(13.00 - 22.60)	(13.20 - 23.00)	(11.22 - 19.56)
Increment QALYs^III^	3.33	3.33	3.33	2.40	−
**Initiation age of 40**					
Remaining life years^I^	20.1	20.1	20.1	19.7	19.6
Costs (US$)^II^	9669.80	9659.97	9731.08	13180.33	13634.36
	(2107.23 - 20052.17)	(2106.35 - 20048.25)	(2109.00 - 20055.12)	(3161.46 - 26225.65)	(4648.40 - 29925.24)
Saving costs(US$)^III^	3964.56	3974.39	3903.28	454.03	−
QALYs ^IV^	15.47	15.46	15.47	14.25	12.88
	(7.96 - 17.56)	(7.76 - 17.56)	(7.96 - 17.56)	(6.84 - 15.64)	(6.59 - 15.19)
Increment QALYs^III^	2.59	2.58	2.59	1.37	−
**Initiation age of 60**					
Remaining life years^I^	7.5	7.5	7.5	7.4	7.4
Costs (US$)^II^	5983.39	5921.23	5928.73	7606.03	8000.42
	(1126.44 - 9436.12)	(1119.18 - 9425.71)	(1119.22 - 9429.12)	(1939.74 - 10312.51)	(2557.1 -16952.10)
Saving costs(US$)^III^	2017.03	2079.19	2071.69	394.39	−
QALYs^IV^	6.32	6.32	6.32	6.09	5.76
	(4.26 - 11.33)	(4.26 - 11.19)	(4.30 - 11.23)	(3.41 - 10.12)	(3.35 - 9.65)
Increment QALYs^III^	0.56	0.56	0.56	0.33	−

^I^: The figures were the mean values of remaining survival years per subject with IGT or diabetes. ^II^: Costs (US$) were the average cost (95% credible intervals) per subject, which arise for subjects from prevention and treatments of diabetes-related disorders during 40 years. ^III^: Saving costs were the average cost per subject in prevention groups in comparison with that of control; Increment QALYs were the average QALYs per subject diagnosed with IGT or diabetes in prevention groups in comparison with that of control. ^IV^: QALYs were the average QALYs per subject with IGT or diabetes (95% credible intervals).

In relation to the economics, the average costs per subject for the controls was $20,103, $13,634 and $8000 at the initiation ages of 25, 40 and 60 years, respectively, which was much higher than that of the prevention strategies within each initiation age. Therefore, screening and lifestyle interventions were associated with greater health benefits at a lower cost relative to no screening. From a societal perspective, these prevention strategies were economical at all initiation ages, especially in the young cohort. However, the differences between interventions were not statistically significant (p >0.9999 within each age group).

The sensitivity analyses showed that the sensitivity of cost savings and increment QALYs to 20% increases or decreases in the main assumptions, such as the screening positive rate, incidence of diabetes and related complications, the total costs of screening, interventions and treatment, all of the utilities associated with diabetes-related disorders and the discount rates. Furthermore, sensitivity of savings concerning the change of the transition rate of IGT, which increased 200% and decreased 50%, was also reported. The comparisons of the four prevention strategies with the control were fairly insensitive for all of these assumptions at the different initiation ages (see Additional file 1). Conversely, by decreasing the detection level of IGT and of the compliance level of screening, the costs per person would increase and the savings would reduce for the prevention strategies. As shown in Table 5, the savings induced by the prevention strategies decreased 50% or more at all initiation ages when the screening compliance rate dropped to 60% (see Additional file 1 for more details).

**Table 5:** **Sensitivity of saving costs (US$**) **per subject to different compliance levels of screening initiated at different ages**

**Different strategies**	**Reference**	**Initiation age of 25**		**Reference**	**Initiation age of 40**		**Reference**	**Initiation age of 60**	
		**80%**	**60%**		**80%**	**60%**		**80%**	**60%**
Screening with diet intervention	6808.10	5057.48	3084.12	3964.56	2885.25	1726.41	2017.03	1095.13	449.56
Screening with exercise intervention	6868.67	5075.73	3091.46	3974.36	2893.45	1716.15	2079.19	1069.45	498.66
Screening with duo-intervention	6861.49	5032.46	3066.46	3903.28	2811.16	1765.19	2071.69	1054.01	489.85
Screening alone	1129.79	820.10	650.75	454.03	369.84	245.84	394.39	174.39	57.75

Data are saving costs (US$) per subject who received prevention strategies when the compliance level of screening were dropped to 80% and 60% respectively. The prevention strategies were still cost-saving at every initiation ages, though the declining level of compliance with screening had a great impact on that reduction of savings for all prevention strategies.

## Discussion

A literature search reveals that the only study of the cost effectiveness for diabetes interventions in an Asian developing country is the Indian Diabetes Prevention Programme [10]. However, this study was only based on 3 years of short-term follow-up and, therefore, does not reflect the long-term economic profile of diabetes prevention. Our study has conducted the first economic analysis of systematic prevention for diabetes in China and includes a large amount of high-quality data on benefits and costs, and also includes various sensitivity analyses.

Significant differences of remaining survival years for individuals with IGT or diabetes were found between the three lifestyle intervention strategies (diet, exercise and duo-intervention) and the other two groups (one-off screening with no intervention strategy and the control) at the initiation ages of 25 and 40, but not 60 years. Consistent with previous studies [12-14,16], our results showed that the extra time for pre-diabetes before diabetes onset was existent, and it was 0.49–2.94 years at the three initiation ages of the simulated intervention strategies. This extra time was less than that found in the USA where it was 6.3 years for prevention initiated at age 30 years, 4.72–5.98 years at age 45 years, and 1.83 years at age 60 years or older [18]. A likely explanation for this difference could be that diabetes was diagnosed much earlier and effective medications were prescribed more widely in higher income countries, while effective management and screening of diabetes and IGT are fairly limited in developing countries [3,46-48]. For the screening alone strategy, the extra time was 0.04 years, which is comparable to the range of 0.02–0.08 reported in the UK and Taiwan [19,20,26]. Consequently, the strategy of screening alone, which results in early monitoring or treatment for diabetes, can improve health [49]. In terms of preventing complications, there is now a broad consensus that the earlier diabetes is detected and treated, the greater the likelihood that complications will be prevented or delayed [27]. Our study has further confirmed that screening and interventions increase the time before developing any complications in IGT subjects.

The estimated costs of screening and lifestyle interventions in this study ($360–$370 per year) are much lower than those of the Diabetes Prevention Programme conducted in the USA ($2780 per year) [11]. This lower cost is primarily because of the following reasons: (i) the personnel costs in developing countries are lower, (ii) our study was based on simulated community interventions, which included group counselling instead of the more costly one-to-one clinical trials, and (iii) the average cost of diabetes intervention in a typical Chinese community was estimated from a later starting period than that of the American programme that started during the initial phase of intervention, which is a more costly period since it included frequent laboratory tests and clinical follow-ups. Although the prevention costs are lower in developing countries, some patients may still be unwilling to pay the costs especially for long-term preventions, such as in China. This is mainly owing to the fact that most Chinese have to pay all prevention expenses out of their own pockets irrespective of the insurance plan that they have [50]. This situation differs from that in many European countries where insurance companies and other health providers cover most costs of the prevention programmes [51].

In the present study, all simulated screening strategies reduced the lifetime costs by approximately $390 or more per screened subject at all the initiation ages as compared with the control; in other words, all the prevention programmes were cost saving. These results are not only considered economically attractive by international standards [21], but are also seen as better than some cost-effective diabetes prevention programmes in high-income countries, such as in Taiwan ($17,113 per QALY gained) [19], Australia ($10,142 per QALY gained) [52], USA ($9731 per QALY gained) [18] and UK ($8358 per QALY gained) [26]. It is noteworthy that the least savings gained by interventions in our study were approximately $2000 per subject among different initiation ages, which is still more than that of some cost-saving countries like Mexico ($1000 saved per subject) [53], Switzerland ($1040 saved per subject) and Germany ($600 saved per subject) [54]. This benefit is due to the long-term effects beyond the intervention period for postponing or averting diabetes and related complications, which bring about substantial high medical costs in China [4,5], even when the interventions were performed for just 6 years.

Among the three simulated intervention programmes, screening with exercise had the greatest savings at all three starting ages compared with the control. However, the differences between these three lifestyle interventions were insignificant. A possible explanation for this could be that the incidences and mortalities of the main diabetes-related complications that we used were the same, since these parameters were calculated after combining the different lifestyle intervention groups [35,36].

Targeting a population at an appropriate age for receiving preventive intervention should be more effective for lowering costs than by targeting a non-specific age. Because the best net health benefits and the greatest saving costs were realized at the age of 25 years, selection of appropriate age groups should not ignore the young adults in developing countries, even though the current recommendations in developed countries are that screening should begin at the age of 45 years [21]. As for older subjects, lifestyle intervention was also observed to result in a reduction of costs and more favourable health consequences, which is similar to the pharmacological interventions [55]. However, these results were not as marked as those for the young and middle-aged, as the older patients with pre-diabetes were not as susceptible to improvements because they responded poorly to a single lifestyle intervention as compared with receiving two or more interventions together [56]. Furthermore, along with the higher incidence and mortality of disease and the significant complications or comorbidities [41,51], the older subjects with IGT were more likely to remain in the death or diabetes-related disorder states since there was no intervention at all in the screening alone and control groups.

Sensitivity analysis found that long-term outcomes were not sensitive to the changes of reference assumptions and they still supported the conclusion, i.e. screening and intervention strategies are cost saving. Because of the chronic and asymptomatic nature of diabetes, screening performance and compliance are important issues for diabetes prevention. Similar to previous studies [26], we found that a lower detection level of IGT from screening and lower compliance with screening resulted in higher costs and lower health effectiveness. However, the prevention programmes may still be worth expanding in China, since they were shown to be cost saving. Meanwhile, maintaining high rates of screening performance and compliance will lead to favourable clinical and economic effects on diabetes prevention.

There were also a few limitations of this study. First, the utilities we used were estimated based on the US population, and they may be on the low side compared with Chinese circumstances [57,58]. Thus, the overall QALYs would increase when the China-specific utilities are available. Nonetheless, the lower utilities might not have significant impact on the stability of the models, since the saving costs were not very sensitive to most changes of utilities.

A second limitation of this study was that the longer life span induced by preventions may result in additional expenditures called ‘future costs’ , which probably should have been considered in the economic analysis [59]. Unfortunately, because of the lack of comprehensive data to estimate the future costs accurately [60], we only included the costs of diabetes prevention and treatments during the simulated 40 years. However, it has been shown that economic analysis, excluding future costs and effects, could still maintain internal consistency [60].

A third limitation was that the influence of repeated screening was not assessed in this study. The saving costs for longer time interval screening ought to be similar to that of one-off screening, while it might decrease for short interval screening, because the prevalence of undiagnosed diabetes and IGT could possibly be lower and result in a higher cost per case [18,20]. The sensitivity analyses suggested that the saving costs of all strategies were insensitive to the prevalence of IGT and diabetes, that is, whether a subject undergoes a repeat screening might not actually affect our results.

Finally, this study did not consider the impact of behavioural or biomarker risk factors such as smoking and haemoglobin A_1c_ concentration [24,51]. Also, the potential benefits of screening and management of related disorders like hypertension and hyperlipidaemia were not taken into account [3,18]. Further investigations of China-specific clinical data with reference to the related subgroups should be explored.

Despite these limitations, the model truly reproduces the effects of diabetes screening and lifestyle interventions. Comprehensive validations that were performed further promoted the accuracy of the model. The findings of this study should be applicable to real lives in China and other developing countries, and it should be able to assist governments of developing countries on strategic decision making regarding health resource allocation over the long term.

## Conclusions

Compared with high-income areas, developing countries are deficient in the effective management of diabetes screening in the general population and in early diagnosis to enable timely IGT interventions, because of insufficient resources and practical considerations. Policies of one-off screening for undiagnosed diabetes and IGT, followed by appropriate lifestyle interventions for those with IGT, are cost saving. These policies represent the effective use of healthcare resources in developing countries, especially when they are applied to young adults.

## Abbreviations

DM: Diabetes mellitus; IGT: Impaired glucose tolerance; OGTT: Oral glucose tolerance test; QALYs: Quality-adjusted life-years; PG: Plasma Glucose; CVD: Cardiovascular disease.

## Competing interests

The authors declare that they have no competing interests.

## Authors’ contributions

LXQ, LCP carried out the study design, participated in the sequence data collection and drafted the manuscript. HG carried out the model analysis and collected data. CZ, FLL and YWH participated in the collected date and performed the statistical analysis. MJ conceived of the study, and participated in its design and also coordinated and helped to draft the manuscript. All authors read and approved the final manuscript.

## Pre-publication history

The pre-publication history for this paper can be accessed here:

http://www.biomedcentral.com/1471-2458/13/729/prepub

## Supplementary Material

Additional file 1 **Sensitivity of saving costs (US$) or increment QALYs to different assumptions for prevention strategies or control.** This file involved the results of sensitivity analyses to different assumptions for different strategies, figures in tables were saving costs (US$) or increment QALYs. The file consisted of four separate sheets. Sheet 1 named “Performance and Compliance” involving the sensitivity of saving costs (US$) and increment QALYs to different compliance and detection level of screening at different initiation ages. The sheet 2–4 named “Initiation age of 25”, “Initiation age of 40” and “Initiation age of 60” respectively, involving the sensitivity of saving costs (US$) or increment QALYs to different assumptions for preventions strategies starting at age of 25, 40 and 60 years. In each sheet, the “OGTT” means oral glucose tolerance test. “IGT” means impaired glucose tolerance. “QALYs” means quality-adjusted life-years. As shown in sheet 1, decreasing the detection level of IGT and of the compliance level of screening would increase the costs per subject, and would result in a reduction of health effectiveness of subjects with diabetes or IGT in prevention groups. Sheet 2–4 showed the sensitivity of saving costs (US$) or increment QALYs to 20% increase or decrease in most assumptions, except the incidence of IGT which increased 200% and decreased 50% at all initiation ages. The insensitive results still supported the main conclusion that screening and intervention strategies for diabetes were cost-saving in China. Click here for file
